# Difference in subjective sleep quality and related lifestyle habits of student-athletes according to chronotype: a cross-sectional study

**DOI:** 10.1186/s13102-025-01151-0

**Published:** 2025-05-13

**Authors:** Takafumi Monma, Goichiro Yoshida, Eiji Fujita, Maki Yamane, Kayoko Ando, Naomi Omi, Hiroyuki Sagayama, Fumi Takeda

**Affiliations:** 1 Institute of Health and Sport Sciences, University of Tsukuba, Tsukuba, Japan; 2 Faculty of Sports and Life Science, National Institute of Fitness and Sports in Kanoya, Kanoya, Japan; 3 Faculty of Sport Sciences, Nihon Fukushi University, Mihama, Japan

**Keywords:** Student-athlete, Chronotype, Subjective sleep quality, Sleep duration, Morning practice

## Abstract

**Background:**

Sleep plays a vital role in the recovery of athletes, yet many student-athletes struggle with poor sleep quality. The literature has reported that chronotypes, which reflect different circadian phases, are related to poor sleep quality and lifestyle habits. However, there is a lack of findings specific to student-athletes. This study aimed to investigate differences in subjective sleep quality and related lifestyle habits among student-athletes based on their chronotype.

**Methods:**

Student-athletes were defined as university athletic club members. A cross-sectional web-based survey collected data from 665 student-athletes at three Japanese universities (male: 70.7%; mean age: 19.7 ± 0.8 years). Chronotypes were assessed using the Morningness-Eveningness Questionnaire, while subjective sleep quality was measured using the Pittsburgh Sleep Quality Index. Lifestyle habits included skipping breakfast, taking caffeinated drinks, using a smartphone/cellphone after lights out, and morning practice. Subjective sleep quality and these lifestyle habits were compared among chronotypes using one-way analysis of covariance (ANCOVA), along with binomial and ordinal logistic regression analyses, adjusted for sex and age.

**Results:**

The chronotype distribution was 15.9% eveningness, 72.9% intermediate, and 11.1% morningness. Individuals with later chronotypes had a higher prevalence of poor sleep quality. Additionally, a higher percentage of those with later chronotypes skipped breakfast and used a smartphone/cellphone after lights out. A lower percentage of later chronotypes also participated in morning practice ≥ 4 days/week. However, morning practice ≥ 4 days/week was associated with poor subjective sleep quality only among student-athletes with an evening chronotype, not among those with intermediate or morning chronotype.

**Conclusion:**

Addressing poor lifestyle habits and promoting earlier chronotypes may be crucial for improving subjective sleep quality in student-athletes with an evening chronotype.

**Supplementary Information:**

The online version contains supplementary material available at 10.1186/s13102-025-01151-0.

## Background

Sleep is crucial for the mental and physical recovery of athletes undergoing intense daily training [1]. However, reports indicate that many student-athletes experience poor sleep quality. Monma et al. [2] found that the percentage of student-athletes with poor subjective sleep quality (defined as > 5 points on the Pittsburgh Sleep Quality Index [PSQI]) was 46.5%, which was higher than the proportion found among elite athletes aged ≥ 20 years (28.0%) in another study that also used the same scale [3]. Therefore, student-athletes require assistance in maintaining their sleep quality.

Understanding circadian rhythms is essential for assessing sleep health. Circadian rhythms are approximately 24-hour biological cycles [4] that regulate the sleep/wake cycle [5]. Chronotype reflects the circadian phase, referring to the biological patterns of circadian rhythms [6]. Alternatively, chronotype can also be assessed based on circadian preference, which reflects individual differences in the timing of various activities, including sleep-wake behavior [7, 8]. A person’s chronotype is primarily determined by genetic factors [9], but individual, environmental, and social aspects also play a role. For example, studies on adolescents have reported that differences in school start times are associated with circadian preference [10]. When considering strategies to improve sleep quality in student-athletes, assessing chronotype through circadian preference is important, as it allows for behavioral and environmental interventions.

Based on preferred sleep habits and activity timing, chronotypes can be classified into morning, intermediate, and evening chronotypes [11]. Previous studies have shown that students and athletes, including student-athletes, with an evening chronotype experience poor sleep quality [12, 13, 14, 15]. A study involving elite Korean athletes examined the components of the PSQI, revealing that individuals with an evening chronotype showed poorer sleep quality, longer sleep latency, more sleep disturbance, and greater daytime dysfunction compared to those with morning or intermediate chronotypes [16]. However, further investigation is warranted, particularly concerning the relationship between chronotypes and subjective sleep quality among student-athletes.

Moreover, lifestyle habits such as skipping breakfast, taking caffeinated drinks, using a smartphone/cellphone after lights out, and engaging in high-frequency morning practices are associated with subjective sleep quality among student-athletes [2, 17]. These habits vary depending on chronotype. For instance, collegiate students with an evening chronotype are more likely to skip breakfast and consume caffeinated drinks in the afternoon or evening [18, 19]. Additionally, increased duration of mobile phone use in bed is positively correlated with the midpoint of sleep among collegiate students [20].

Certain athletic populations regularly participate in morning practice sessions; however, frequent morning training may have adverse effects on sleep [2]. Considering chronotypes, individuals with an evening chronotype typically go to bed and wake up later [11]. Consequently, many student-athletes with an evening chronotype may not frequently engage in morning practices, but those who do them frequently may have poor sleep quality. However, it remains unclear whether lifestyle habits vary by chronotype and whether the relationship between morning practices and subjective sleep quality differs depending on the chronotype of student-athletes.

Furthermore, differences in sports disciplines may be associated with these relationships. Previous studies have reported that athletes in individual sports tend to have a more morning-oriented chronotype [21], with earlier bedtimes and wake-up times [21, 22] and shorter sleep duration [22] than those in team sports. Given these differences in sleep habits, the relationship between subjective sleep quality, lifestyle habits, and chronotype may vary depending on the sports discipline. However, this aspect has not yet been examined.

Therefore, this study aimed to elucidate differences in subjective sleep quality and associated lifestyle habits based on the chronotypes of student-athletes.

## Methods

### Procedure and participants

In this study, “student-athletes” were defined as individuals affiliated with university athletic clubs. This study targeted 1175 students who attended the classes for sophomores and junior students in three Japanese universities’ physical education faculties from three Japanese regions. These students were asked during class to cooperate in a web-based questionnaire survey conducted in June 2022. They completed the survey within one week at a location of their choice. The number of surveys recovered was 841 (a recovery rate of 71.6%). While students who were not sophomores and junior students and did not belong to a university athletic club also attended these classes, their data were excluded. Thus, 690 respondents remained; and from these, 665 respondents with complete response were selected for analysis (valid response rate: 96.4%).

### Questionnaires

The questionnaire included attributes, chronotype, sleep health, and other lifestyle habits. Attributes included sex, age, and competitive events.

Chronotype classification utilized the Japanese version of the Morningness-Eveningness Questionnaire (MEQ) [23, 24], featuring 19 items determining preferred times for habitual physical and mental activities. MEQ scores, ranging from 16 to 86, categorized individuals into definitely evening type (16–30), moderately evening type (31–41), intermediate type (42–58), moderately morning type (59–69), and definitely morning type (70–86).

Regarding sleep health, subjective sleep quality was assessed using the Japanese version of the PSQI [25, 26]. The PSQI is the most commonly used scale for measuring subjective sleep quality [27]. This scale comprises 18 questions on sleep habits over the previous month. Seven component scores were derived: “sleep quality,” “sleep latency,” “sleep duration,” “habitual sleep efficiency,” “sleep disturbances,” “use of sleep medication,” and “daytime dysfunction.” Each component was scored on a scale of 0–3, with higher scores indicating worse sleep conditions. The global PSQI score ranged from 0 to 21 points, with scores > 5 indicating “poor sleep quality” (when used to screen for insomnia, sensitivity was 85.7%, while specificity was 86.6% [26]). Additionally, sleep habits such as sleep timing (bedtime, waking time, and midpoint of sleep) and sleep duration, commonly used as sleep health measures [28, 29], were collected from the PSQI responses.

Other lifestyle habits, known to be related to subjective sleep quality in student-athletes [2, 17], were examined. These included skipping breakfast, taking caffeinated drinks, using a smartphone/cellphone after lights out, and morning practice. For skipping breakfast, the frequency of skipping per week was assessed. Taking caffeinated drinks before bed was evaluated with six options (every day, 5–6 days/week, 3–4 days/week, 1–2 days/week, 1–3 days/month, and none). The use of smartphones/cellphones after lights out was assessed using a dichotomized scale (yes/no). Regarding morning practice frequency, respondents reported the number of days per week they had practice at 9:00 a.m. or earlier as part of their regular training routine. Whether the practice was mandatory or voluntary was not considered.

### Statistical analysis

The participants were categorized into three chronotypes: “eveningness” (definitely evening type and moderately evening type), “intermediate,” and “morningness” (moderately morning type and definitely morning type). Sleep health and other lifestyle habits were compared among chronotypes through one-way analysis of covariance (ANCOVA), along with binomial and ordinal logistic regression analyses, adjusted for age and sex, as sleep health varies across these factors [30]. Multiple comparisons (using the Bonferroni method) were conducted in ANCOVA for variables exhibiting significant differences among chronotypes. Morning chronotype served as the reference category in binomial and ordinal logistic regression analyses. To control the false discovery rate (FDR), the Benjamini–Hochberg procedure was employed for adjusting *p*-values in three sets of analyses: (1) bedtime, wake-up time, midpoint of sleep, and sleep duration; (2) the seven component scores of the PSQI; and (3) other lifestyle habits. Subsequently, chi-square and Fisher’s exact tests were employed to explore the association between morning practices and subjective sleep quality (poor/good) across each chronotype. Finally, after comparing the chronotypes, sleep health, and lifestyle habits by sports discipline (individual or team sports), the above analyses were conducted by classifying sports disciplines. Some competitive events have both individual and team disciplines, but they were classified according to the predominant element of the sport as a whole.

Following previous research [2], lifestyle habits were categorized as: skipping breakfast – “yes” (more than once a week) and “no”; taking caffeinated drinks – “yes” (more than once a month) and “no”; morning practice – “‘0–3 days per week” and “4–7 days per week.” Statistical analyses were performed using SPSS Statistics 28.0 for Windows, with a significance level set at 5%.

## Results

Table 1 displays respondents’ attributes, morning practices, sleep health, and other lifestyle habits. Of the participants, 70.7% were male, with a mean age of 19.7 ± 0.8 years. On average, the PSQI score stood at 4.96 ± 2.47, with 252 respondents (37.9%) indicating poor sleep quality based on this score. Table 2 illustrates the breakdown of respondents involved in competitive events, with 111 (16.7%) in track and field, 94 (14.1%) in soccer, 85 (12.8%) in baseball, 58 (8.7%) in kendo, and 47 (7.1%) in basketball.

**Table 1:** Respondents’ characteristics

					*N* (%) or Mean ± SD or Median [QD]		
Attributes							
	Sex			Male	470	(70.7)	
				Female	195	(29.3)	
	Age (years)				19.7	±	0.8
Sleep Health							
	Bedtime (hour)				0:02	±	1:07
	Wake-up time (hour)				7:29	±	1:20
	Midpoint of sleep (hour)				3:46	±	1:04
	Sleep duration (hour)				6:46	±	1:04
	Subjective sleep quality						
		Global PSQI score (points)			4.96	±	2.47
		Poor sleep quality		Presence	252	(37.9)	
				Absence	413	(62.1)	
		Component score (points)					
			Sleep quality		1.00	[0.50]	
			Sleep latency		1.00	[1.00]	
			Sleep duration		1.00	[1.00]	
			Sleep efficiency		0.00	[0.00]	
			Sleep disturbance		1.00	[0.50]	
			Use of sleep medication		0.00	[0.00]	
			Daytime dysfunction		1.00	[0.50]	
Lifestyle habits							
	Skipping breakfast			Yes	361	(54.3)	
				No	304	(45.7)	
	Taking caffeinated drinks			Yes	312	(46.9)	
				No	353	(53.1)	
	Using smartphone/cellphone after lights out			Yes	454	(68.3)	
				No	211	(31.7)	
	Morning practice			0–3 days/week	530	(79.7)	
				4–7 days/week	135	(20.3)	

SD: Standard deviation, QD: Quartile deviation; PSQI: Pittsburgh Sleep Quality Index

**Table 2:** Distribution of respondents by competitive events

	*N* (%)	
Track and field	111	(16.7)
Soccer	94	(14.1)
Baseball	85	(12.8)
Kendo	58	(8.7)
Basketball	47	(7.1)
Swimming	39	(5.9)
Tennis	30	(4.5)
Judo	28	(4.2)
Rugby	25	(3.8)
Volleyball	22	(3.3)
Badminton	18	(2.7)
Gymnastics	15	(2.3)
Softball	13	(2.0)
Canoe	10	(1.5)
Handball	9	(1.4)
Dance	9	(1.4)
Table tennis	8	(1.2)
Bicycling	6	(0.9)
Water polo	6	(0.9)
Lacrosse	5	(0.8)
American football	5	(0.8)
Japanese archery	4	(0.6)
Naginata	3	(0.5)
Rowing	3	(0.5)
Archery	3	(0.5)
Sailing	2	(0.3)
Windsurfing	1	(0.2)
Ice hockey	1	(0.2)
Artistic swimming	1	(0.2)
Diving	1	(0.2)
Aerobic	1	(0.2)
Shorinji kenpo	1	(0.2)
Skeleton	1	(0.2)

**Fig. 1:**
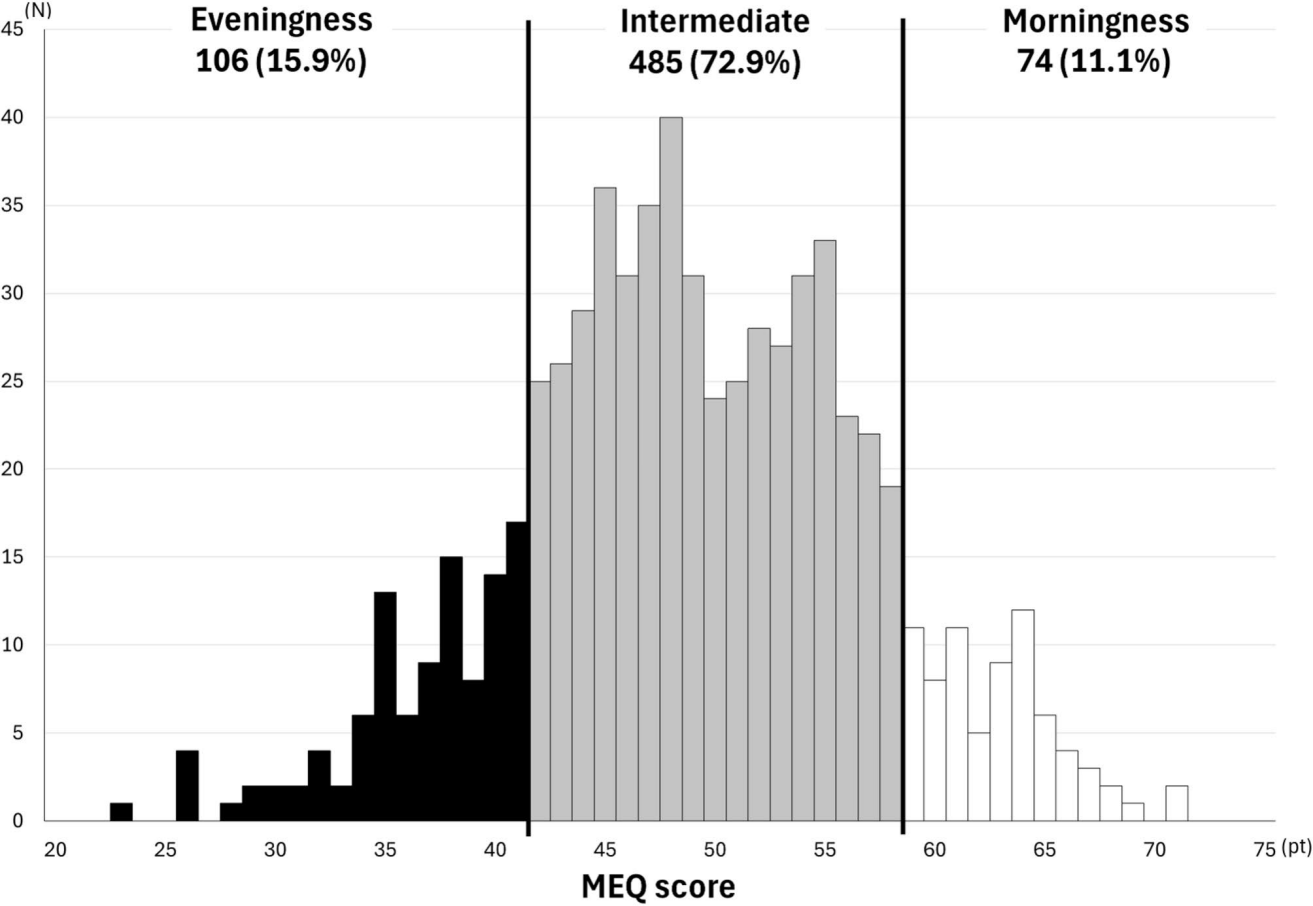
Respondents’ chronotype

Figure 1 shows the distribution of chronotypes. The mean MEQ score was 49.0 ± 8.1 points. Of the respondents, 106 (15.9%) were evening chronotypes, 485 (72.9%) were intermediate types, and 74 (11.1%) were morning chronotypes.

Table 3 presents a comparison of sleep habits across different chronotypes. Significant differences were observed in bedtime, waking time, and midpoint of sleep based on chronotype. The evening chronotype exhibited the latest bedtime, waking time and midpoint of sleep, followed by intermediate and morning chronotypes.

**Table 3:** Comparison of sleep habits between the chronotypes

	Morningness			Intermediate			Eveningness					Multiple comparisons
	Mean	±	SD	Mean	±	SD	Mean	±	SD	F		
Bedtime (hour)	23:18	±	1:03	0:02	±	1:00	0:54	±	1:08	59.01	*	E > I > M
Wake-up time (hour)	6:35	±	1:02	7:25	±	1:14	8:26	±	1:22	51.11	*	E > I > M
Midpoint of sleep (hour)	2:56	±	0:53	3:42	±	0:57	4:40	±	1:05	74.31	*	E > I > M
Sleep duration (hour)	6:43	±	1:05	6:47	±	1:04	6:39	±	1:05	0.97		

E: Eveningness, I: Intermediate, M: Morningness

Analysis of covariance adjusted for sex and age

* *P* value was statistically significant after applying the Benjamini–Hochberg false discovery rate (FDR) correction

Table 4 shows the results of the comparison between subjective sleep quality and the seven components. The proportion of poor sleep quality was higher in the later chronotype and statistically higher for the evening and intermediate chronotypes than for the morning chronotype. Regarding the component scores, the sleep quality and sleep latency scores for the evening and intermediate chronotypes were statistically higher than those for the morning chronotype. Sleep efficiency and daytime dysfunction scores were significantly higher for the evening chronotype than for the morning chronotype.

**Table 4:** Comparison of subjective sleep quality between the chronotypes

			Morningness		Intermediate							Eveningness						
			N	(%)	N	(%)	AOR		95%CI			N	(%)	AOR		95%CI		
Subjective sleep quality		Absence	57	(77.0)	309	(63.7)	1.96	*	1.10	-	3.48	47	(44.3)	4.40	*	2.25	-	8.58
		Presence	17	(23.0)	176	(36.3)						59	(55.7)					
Component scores^a^																		
	Sleep quality	0 points	11	(14.9)	47	(9.7)	1.87	*	1.14	-	3.07	5	(4.7)	3.32	*	1.83	-	6.03
		1 point	48	(64.9)	277	(57.1)						53	(50.0)					
		2 points	14	(18.9)	151	(31.1)						43	(40.6)					
		3 points	1	(1.4)	10	(2.1)						5	(4.7)					
	Sleep latency	0 points	37	(50.0)	163	(33.6)	1.87	*	1.18	-	2.97	25	(23.6)	3.80	*	2.17	-	6.65
		1 point	24	(32.4)	197	(40.6)						35	(33.0)					
		2 points	11	(14.9)	97	(20.0)						31	(29.2)					
		3 points	2	(2.7)	28	(5.8)						15	(14.2)					
	Sleep duration	0 points	21	(28.4)	143	(29.5)	1.00		0.64	-	1.57	26	(24.5)	1.40		0.81	-	2.42
		1 point	30	(40.5)	188	(38.8)						38	(35.8)					
		2 points	21	(28.4)	143	(29.5)						38	(35.8)					
		3 points	2	(2.7)	11	(2.3)						4	(3.8)					
	Sleep efficiency	0 points	62	(83.8)	370	(76.3)	1.60		0.84	-	3.07	71	(67.0)	2.60	*	1.25	-	5.41
		1 point	9	(12.2)	88	(18.1)						25	(23.6)					
		2 points	3	(4.1)	21	(4.3)						6	(5.7)					
		3 points	0	(0.0)	6	(1.2)						4	(3.8)					
	Sleep disturbance	0 points	34	(45.9)	172	(35.5)	1.58		0.96	-	2.59	39	(36.8)	1.54		0.84	-	2.81
		1 point	39	(52.7)	305	(62.9)						65	(61.3)					
		2 points	1	(1.4)	8	(1.6)						2	(1.9)					
	Using sleep medication	0 points	72	(97.3)	476	(98.1)	0.65		0.14	-	3.13	106	(100.0)	0.00		0.00		
		1 point	1	(1.4)	5	(1.0)						0	(0.0)					
		2 points	1	(1.4)	4	(0.8)						0	(0.0)					
	Daytime dysfunction	0 points	43	(58.1)	239	(49.3)	1.41		0.87	-	2.29	40	(37.7)	2.23	*	1.25	-	3.99
		1 point	25	(33.8)	202	(41.6)						53	(50.0)					
		2 points	5	(6.8)	39	(8.0)						10	(9.4)					
		3 points	1	(1.4)	5	(1.0)						3	(2.8)					

AOR: adjusted odds ratio, CI: confidence interval

Analysis adjusted for age and sex

*: *P*-value was statistically significant

^a^: *P*-value was adjusted by Benjamini-Hochberg false discovery rate (FDR) correction

In the comparisons of lifestyle habits between the chronotypes (Table 5), the proportions of skipping breakfast and using a smartphone/cellphone after lights out were significantly higher in the evening and intermediate chronotypes than in the morning chronotype. In contrast, the proportion of morning practice 4–7 days/week was significantly lower in the evening and intermediate chronotypes than in the morning chronotype.

**Table 5:** Comparison of lifestyle habits among the chronotypes

		Morningness		Intermediate							Eveningness						
		N	(%)	N	(%)	AOR		95%CI			N	(%)	AOR		95%CI		
Skipping breakfast	Yes	13	(17.6)	261	(53.8)	5.79	*	3.08	-	10.89	87	(82.1)	23.55	*	10.72	-	51.74
	No	61	(82.4)	224	(46.2)						19	(17.9)					
Taking caffeinated drinks	Yes	27	(36.5)	235	(48.5)	1.64		0.99	-	2.72	50	(47.2)	1.56		0.85	-	2.88
	No	47	(63.5)	250	(51.5)						56	(52.8)					
Using smartphone/cellphone after lights out	Yes	37	(50.0)	333	(68.7)	2.23	*	1.36	-	3.66	84	(79.2)	3.96	*	2.05	-	7.65
	No	37	(50.0)	152	(31.3)						22	(20.8)					
Morning practice	4–7 days/week	26	(35.1)	103	(21.2)	0.49	*	0.29	-	0.83	6	(5.7)	0.11	*	0.04	-	0.28
	0–3 days/week	48	(64.9)	382	(78.8)						100	(94.3)					

AOR: adjusted odds ratio, CI: confidence interval

Analysis adjusted for age and sex

* *P* value was statistically significant after applying the Benjamini–Hochberg false discovery rate (FDR) correction

Table 6 shows the relationship between morning practice and sleep quality according to the chronotype. In terms of evening chronotype, respondents who practiced 4–7 days/week in the morning were more likely to have poor sleep quality than those who practiced 0–3 days/week.

**Table 6:** Relationships between morning practice and subjective sleep quality by chronotype

		Morningness						Intermediate						Eveningness					
		Subjective sleep quality																	
		Poor		Good				Poor		Good				Poor		Good			
		(*N* = 17)		(*N* = 57)		*P*		(*N* = 176)		(*N* = 309)		*P*		(*N* = 59)		(*N* = 47)		*P*	
Morning practice	0–3 days/week	11	(64.7)	37	(64.9)	1.000	^a^	139	(79.0)	243	(78.6)	0.931	^a^	53	(89.8)	47	(100.0)	0.033	^b^
	4–7 days/week	6	(35.3)	20	(35.1)			37	(21.0)	66	(21.4)			6	(10.2)	0	(0.0)		

^a^Chi-square test

^b^Fisher’s exact test

The results of the analysis stratified by sports discipline are shown in Supplementary Tables 1–5. No differences in chronotypes, sleep health, and lifestyle habits were observed between individual and team sports (Supplementary Table 1). Regarding sleep habits, for both individual and team sports, the evening chronotype exhibited the latest bedtime, waking time, and midpoint of sleep, followed by the intermediate and morning chronotypes (Supplementary Table 2). Additionally, in individual and team sports, poor sleep quality was statistically higher for the evening chronotype than for the morning chronotype (Supplementary Table 3). Regarding the component scores, the sleep latency score was significantly different by chronotype for both individual and team sports. Sleep quality and efficiency scores showed significant differences by chronotype only for individual sports (Supplementary Table 3). Regarding lifestyle habits, the proportions of respondents who skipped breakfast, used a smartphone/cellphone after lights out, and did morning practice 4–7 days/week were significantly different between the chronotypes for both individual and team sports (Supplementary Table 4). Regarding the relationship between morning practice and sleep quality according to chronotype, no significant relationships were found for individual or team sports (Supplementary Table 5).

## Discussion

The chronotype distribution among the respondents in this study was as follows: 15.9% eveningness, 72.9% intermediate, and 11.1% morningness. In a previous study using the same scale reported, involving 340 athletes (mean age, 17.6 years) in South Korea, the chronotype distribution was as follows: 47.9% eveningness, 48.2% intermediate, and 3.8% morningness [16]. Another study of 88 male (mean age: 21.9 years) and 26 female (mean age: 22.6 years) athletes in Australia reported a distribution of 6.1% eveningness, 64.9% intermediate, and 28.9% morningness [31]. The distribution of chronotypes in the population around the age of 20 years varied notably between studies, and more athletes in the present study had the intermediate chronotype.

In our study, 37.9% of the respondents had poor sleep quality. Although findings on non-athletes of the same age group in Japan vary, large population studies have reported that the proportion of individuals classified as having poor sleep quality ranges from 20.4 to 33.3% [32, 33]. Therefore, student-athletes may have a high prevalence of poor subjective sleep quality. Regarding sleep timing, the average bedtime, wake-up time, and midpoint of sleep among the respondents in our study were 0:02, 7:29, and 3:46, respectively. Compared to data from individuals aged 20–24 years in a Japanese nationally representative survey [34], bedtime and wake-up time were 18 min earlier, respectively. These results may reflect a characteristic of athletes, as a certain proportion engage in morning practice. On the other hand, the average sleep duration among the participants in this study was 6 h and 46 min, over 40 min shorter than that reported among Japanese teens and those in their twenties in a Japanese nationally representative survey [35]. Previous studies have shown that student-athletes tend to have shorter sleep durations than their non-athlete peers of the same age [36], and similar results were observed in our study.

The proportion of respondents with poor sleep quality was higher in the later chronotypes. This result supports previous findings pertaining to college students [12], athletes [13, 14], and student-athletes during the COVID-19 pandemic [15]. In addition, regarding components of PSQI, student-athletes with the evening chronotype had poor sleep quality, sleep latency, low sleep efficiency, and daytime dysfunction. However, sleep duration did not differ according to chronotype. In a Korean study, elite athletes with an evening chronotype reported worse sleep quality, sleep latency, sleep difficulty, and daytime dysfunction [16], similar to the results of the present study. Thus, chronotype may be related to qualitative rather than quantitative aspects of sleep.

One of the reasons for the poor subjective sleep quality in student-athletes with an evening chronotype is likely to be unhealthy lifestyle habits. In this study, the proportions of respondents who skipped breakfast and used smartphones/cellphones after lights out were higher in the later chronotypes. These results are consistent with the findings of previous studies involving college students [18, 20], suggesting that these lifestyle habits can be considered a common characteristic among individuals with an evening chronotype in this age group. These lifestyle habits are associated with poor subjective sleep quality in student-athletes [2, 17]. Therefore, individuals with an evening chronotype may experience poorer subjective sleep quality owing to skipping breakfast and using smartphones/cellphones after lights out.

Another reason behind the relationship between chronotype and subjective sleep quality may be the need for balancing academic and athletic commitments. Student-athletes with an evening chronotype had later bedtimes, wake-up times, and midpoint sleep times than those with morning and intermediate chronotypes. They also had a lower proportion of high-frequency morning practice engagement. However, their academic schedules may lack flexibility and do not align with study rhythms favorable to student-athletes with an evening chronotype. This misalignment may negatively impact their sleep quality.

Furthermore, social jetlag may also influence the relationship between chronotype and subjective sleep quality. Social jetlag is described as a habitual mismatch between circadian rhythms and social schedule [37]. Social jetlag is most prominent in individuals with an evening chronotype [38, 39]. Social jetlag is associated with subjective sleep quality in young individuals [40]. Therefore, preventing social jetlag may help avoid poor subjective sleep quality in student-athletes with an evening chronotype.

The relationship between morning practice and chronotype revealed in this study may suggest the involvement of social jetlag. In this study, the proportion of respondents engaging in morning practice ≥ 4 days/week, which was related to subjective sleep quality, was lower in the later chronotypes. However, in the evening chronotype group, we found a relationship between morning practice ≥ 4 days/week and subjective sleep quality. Although the previous study reported that morning practice ≥ 4 days/week was associated with poor subjective sleep quality in student-athletes, high-frequency morning practice may only be a problem for those with evening chronotype.

In this context, individuals with an evening chronotype appear to experience performance difficulties due to the effects of social jetlag. Among adolescent male basketball players, free throw shooting accuracy in evening types gradually decreased from Monday to Friday but not during holidays, suggesting the impact of social jetlag [41]. Additionally, social jetlag affects brain areas involved in postural control, such as the thalamus, prefrontal cortex, and cerebellum, leading to poorer postural control performance [42]. Recent animal experiments suggest that social jetlag may also impact exercise-induced training adaptations. Mice experiencing social jetlag during voluntary exercise exhibited increased fasting blood glucose levels and impaired exercise performance compared to trained mice not exposed to social jetlag [43].

Furthermore, sleep deprivation seems to negatively affect the athletic performance of student-athletes with an evening chronotype during morning practice. A previous study on Japanese collegiate golfers reported that the evening chronotype was associated with poor golf putting in the morning when the sleep hours were restricted [44]. Considering this finding, frequent morning practice for student-athletes with an evening chronotype may not only negatively impact sleep quality but also be inefficient in terms of athletic performance. Therefore, those student-athletes are recommended to adjust their practice schedules to avoid high-frequency morning practices.

Our analyses of differences in subjective sleep quality and associated lifestyle habits based on chronotypes by sports discipline showed almost identical results for individual and team sports. Our findings suggest that sports discipline has little influence on the differences in subjective sleep quality and associated lifestyle habits based on chronotypes. We found no significant differences in chronotype and sleep health between individual and team sports, although previous studies have reported that athletes in individual sports tend to have a more morning-oriented chronotype [21], with earlier bedtimes and wake-up times [21, 22] and shorter sleep duration [22] than those in team sports. Therefore, further evidence needs to be accumulated, as the characteristics of chronotype and sleep health may have influenced the results of this study. In addition, the relationship between frequent morning practice and poor sleep quality in individuals with an evening chronotype was not observed in either individual or team sports. This result may be attributed to the fact that a very small number of individuals with an evening chronotype practiced morning training 4–7 days a week. Further investigations with larger sample sizes are needed to examine these relationships.

The first step in addressing subjective sleep quality of student-athletes with the evening chronotype is to improve lifestyle habits such as avoiding skipping breakfast and using a smartphone/cellphone after lights out. In addition, adjustment of the circadian rhythm may also be an effective measure for improving sleep. Controlling light exposure is an effective way to regulate the circadian rhythm. Previous studies have reported that morning light exposure advances circadian rhythms, whereas light exposure in the evening or later delays circadian rhythms [45, 46]. Another study reported that blocking short-wavelength light in the evening, compared with habitual light exposure, significantly shortened subjective sleep-onset latency, improved sleep quality, and increased alertness the following morning [47]. Based on these findings, continuous light exposure in the morning and reduced light exposure in the evening can advance circadian rhythms and improve sleep quality.

Additionally, adjusting the training schedule is also important. Based on the findings of this study, student-athletes with an evening chronotype would benefit from avoiding high-frequency morning practices. Moreover, other studies on adolescent athletes [48] and student-athletes [49] have suggested that high-intensity training in the evening may lead to a decline in sleep quality. Therefore, adjusting training schedules based on chronotype may be a key factor in improving sleep quality and promoting the recovery process in student-athletes.

From the perspective of athlete conditioning, particularly in preventing overtraining syndrome, how chronotype modifies the adverse effect of poor sleep quality on health must be examined. Sleep deprivation has been suggested to have an impact on the immune system by activating lymphocytes and increasing the production of inflammatory cytokines, including IL-1β, IL-6, and IL-17 [50]. One study showed that in populations with short sleep duration, a saturation effect is evident in the relationship between physical activity and systemic immune inflammation index, meaning that while moderate physical activity can help reduce inflammation levels, excessive exercise may not provide additional anti-inflammatory benefits [51]. In the future, further exploration is needed to understand how chronotype influences the relationship between sleep deprivation and health in athletes undergoing intense daily training. Additionally, as a practical intervention, studies on how improving sleep quality and adjusting training volume based on chronotype can mitigate adverse health effects may contribute to developing effective athlete conditioning strategies.

This study had several limitations. First, as this was a cross-sectional study, the causal relationship between subjective sleep quality, morning practice, and chronotype should be clarified in future longitudinal studies. Second, the data were collected using self-report questionnaires; therefore, reporting bias cannot be ruled out. Further studies using objective evaluations based on physical and physiological indices are needed. Third, other factors may need to be considered when understanding differences in subjective sleep quality and lifestyle habits based on chronotype. In particular, social jetlag may be strongly related to these differences. Finally, this study found that morning practice ≥ 4 days/week was associated with poor subjective sleep quality only among student-athletes with an evening chronotype. However, since the number of participants with an evening chronotype who engaged in morning practice ≥ 4 days/week was small, further studies with larger sample sizes are needed to examine these relationships.

Despite these limitations, this study is valuable because it details the relationship between chronotype and subjective sleep quality in student-athletes. The significance of this study is that it revealed that student-athletes with the evening chronotype were more likely to have subjective sleep quality problems and poor lifestyle habits. Furthermore, since this study included student-athletes from 33 different disciplines at three universities, the findings can be generalized to student-athletes.

## Conclusions

Student-athletes with a later chronotype are more likely to have poorer subjective sleep quality, skip breakfast, and use their smartphone/cellphone for longer after lights out. Additionally, those with a later chronotype tend to have a lower frequency of morning practice. However, those with an evening chronotype who frequently engage in morning practice appear to have poorer subjective sleep quality. These findings indicate that addressing lifestyle habits and considering chronotype adjustments may contribute to better sleep health in student-athletes with an evening chronotype.

## Electronic supplementary material

Below is the link to the electronic supplementary material.

Supplementary Material 1

## Data Availability

The datasets used and analyzed during the current study are available from the corresponding author on reasonable request.

## Abbreviations

MEQ: Morningness eveningness questionnaire
PSQI: Pittsburgh sleep quality index
ANCOVA: Analysis of covariance
FDR: False discovery rate
