# Supplementary material for: Difference in subjective sleep quality and related lifestyle habits of student-athletes according to chronotype: a cross-sectional study
Source: BMC Sports Sci Med Rehabil. 2025 May 13;17:122. doi: 10.1186/s13102-025-01151-0 (PMC12076887; doi:10.1186/s13102-025-01151-0)
Supplement: Supplementary file 1 — Supplementary Material 1 [file 13102_2025_1151_MOESM1_ESM.docx]

**Supplementary Table 1** Chornotype, sleep health, and lifestyle habits by sports disciplines

|  |  |  |  |  | Individual sports | | | Team sports | | |  |  |
| --- | --- | --- | --- | --- | --- | --- | --- | --- | --- | --- | --- | --- |
|  |  |  |  |  | N (%) or  Mean ± SD or Median [QD] | | | N (%) or  Mean ± SD or Median [QD] | | | *P* | |
| Chronotype | |  |  |  |  |  | |  |  |  |  |  |
|  | Morningness | | |  | 29 | (9.3) | | 45 | (12.7) | | 0.266 | ^a^ |
|  | Intermediate | | |  | 236 | (75.6) | | 249 | (70.5) | |  |  |
|  | Eveningness | | |  | 47 | (15.1) | | 59 | (16.7) | |  |  |
| Sleep health | |  |  |  |  |  |  |  |  |  |  |  |
|  | Bedtime (hour) | | |  | 0:01 | ± | 1:10 | 0:04 | ± | 1:02 | 0.338 | ^b^ |
|  | Wake-up time (hour) | | |  | 7:30 | ± | 1:18 | 7:29 | ± | 1:22 | 0.448 | ^b^ |
|  | Midpoint of sleep (hour) | | |  | 3:45 | ± | 1:06 | 3:46 | ± | 1:03 | 0.446 | ^b^ |
|  | Sleep duration (hour) | | |  | 6:46 | ± | 1:02 | 6:45 | ± | 1:07 | 0.454 | ^b^ |
|  | Subjective sleep quality | | |  |  |  |  |  |  |  |  |  |
|  |  | Global PSQI score (points) | |  | 5.08 | ± | 2.46 | 4.81 | ± | 2.47 | 0.162 | ^b^ |
|  |  | Poor sleep quality | | Presence | 109 | (34.9) | | 143 | (40.5) | | 0.139 | ^a^ |
|  |  |  |  | Absence | 203 | (65.1) | | 210 | (59.5) | |  |  |
|  |  | Component score (points) | |  |  |  | |  |  |  |  |  |
|  |  |  | Sleep quality |  | 1.00 | [0.50] | | 1.00 | [0.50] | | 0.257 | ^c^ |
|  |  |  | Sleep latency |  | 1.00 | [1.00] | | 1.00 | [1.00] | | 0.175 | ^c^ |
|  |  |  | Sleep duration |  | 1.00 | [1.00] | | 1.00 | [1.00] | | 0.884 | ^c^ |
|  |  |  | Sleep efficiency |  | 0.00 | [0.00] | | 0.00 | [0.38] | | 0.719 | ^c^ |
|  |  |  | Sleep disturbance |  | 1.00 | [0.50] | | 1.00 | [0.50] | | 0.137 | ^c^ |
|  |  |  | Use of sleep medication |  | 0.00 | [0.00] | | 0.00 | [0.00] | | 0.268 | ^c^ |
|  |  |  | Daytime dysfunction |  | 1.00 | [0.50] | | 0.00 | [0.50] | | 0.125 | ^c^ |
| Lifestyle habits | | |  |  |  |  | |  |  |  |  |  |
|  | Skipping breakfast | | | Yes | 158 | (50.6) | | 203 | (57.5) | | 0.076 | ^a^ |
|  |  |  |  | No | 154 | (49.4) | | 150 | (42.5) | |  |  |
|  | Taking caffeinated drinks | | | Yes | 147 | (47.1) | | 165 | (46.7) | | 0.923 | ^a^ |
|  |  |  |  | No | 165 | (52.9) | | 188 | (53.3) | |  |  |
|  | Using smartphone/cellphone after lights out | | | Yes | 216 | (69.2) | | 238 | (67.4) | | 0.676 | ^a^ |
|  |  |  |  | No | 96 | (30.8) | | 115 | (32.6) | |  |  |
|  | Morning practice | | | 0–3 days/week | 245 | (78.5) | | 285 | (80.7) | | 0.500 | ^a^ |
|  |  |  |  | 4–7 days/week | 67 | (21.5) | | 68 | (19.3) | |  |  |

SD: Standard deviation, QD: Quartile deviation; PSQI: Pittsburgh Sleep Quality Index

^a^Chi-square test

^b^t-test

^c^Mann-Whitney U-test

**Supplementary Table 2** Comparison of sleep habits between the chronotypes by sports disciplines

|  |  | Morningness | | | Intermediate | | | Eveningness | | |  |  | Multiple comparisons |
| --- | --- | --- | --- | --- | --- | --- | --- | --- | --- | --- | --- | --- | --- |
|  |  | Mean | ± | SD | Mean | ± | SD | Mean | ± | SD | F |  |  |
| Individual sports | |  |  |  |  |  |  |  |  |  |  |  |  |
|  | Bedtime (hour) | 23:11 | ± | 1:01 | 23:58 | ± | 1:04 | 0:53 | ± | 1:11 | 34.20 | * | E>I>M |
|  | Wake-up time (hour) | 6:37 | ± | 0:58 | 7:25 | ± | 1:10 | 8:30 | ± | 1:25 | 34.53 | * | E>I>M |
|  | Midpoint of sleep (hour) | 2:54 | ± | 0:51 | 3:42 | ± | 0:58 | 4:41 | ± | 1:07 | 46.01 | * | E>I>M |
|  | Sleep duration (hour) | 6:53 | ± | 1:00 | 6:46 | ± | 1:02 | 6:42 | ± | 1:03 | 0.55 |  |  |
| Team sports | |  |  |  |  |  |  |  |  |  |  |  |  |
|  | Bedtime (hour) | 23:28 | ± | 1:08 | 23:58 | ± | 0:55 | 0:56 | ± | 1:06 | 25.39 | * | E>I>M |
|  | Wake-up time (hour) | 6:32 | ± | 1:10 | 7:25 | ± | 1:18 | 8:21 | ± | 1:19 | 18.09 | * | E>I>M |
|  | Midpoint of sleep (hour) | 3:00 | ± | 0:56 | 3:41 | ± | 0:57 | 4:38 | ± | 1:03 | 28.60 | * | E>I>M |
|  | Sleep duration (hour) | 6:28 | ± | 1:10 | 6:50 | ± | 1:07 | 6:35 | ± | 1:07 | 2.02 |  |  |

E: eveningness, I: intermediate, M: morningness

Analysis of covariance adjusting for sex and age.

*: P-value was statistically significant after applying Benjamini-Hochberg false discovery rate (FDR) correction.

**Supplementary Table 3** Comparison of subjective sleep quality between the chronotypes by sports disciplines

|  |  |  |  | Morningness | | Intermediate | | | | | | | Eveningness | | | | | | |
| --- | --- | --- | --- | --- | --- | --- | --- | --- | --- | --- | --- | --- | --- | --- | --- | --- | --- | --- | --- |
|  |  |  |  | N | (%) | N | (%) | AOR |  | 95%CI | | | N | (%) | AOR |  | 95%CI | | |
| Individual sports | | |  |  |  |  |  |  |  |  |  |  |  |  |  |  |  |  |  |
|  | Subjective sleep quality | | Absence | 34 | (75.6) | 153 | (61.4) | 2.06 |  | 0.99 | - | 4.29 | 23 | (39.0) | 5.25 | * | 2.20 | - | 12.52 |
|  |  |  | Presence | 11 | (24.4) | 96 | (38.6) |  |  |  |  |  | 36 | (61.0) |  |  |  |  |  |
|  | Component scores^a^ | |  |  |  |  |  |  |  |  |  |  |  |  |  |  |  |  |  |
|  |  | Sleep quality | 0 points | 7 | (15.6) | 17 | (6.8) | 2.88 | * | 1.48 | - | 5.62 | 4 | (6.8) | 5.18 | * | 2.31 | - | 11.60 |
|  |  |  | 1 point | 31 | (68.9) | 144 | (57.8) |  |  |  |  |  | 25 | (42.4) |  |  |  |  |  |
|  |  |  | 2 points | 7 | (15.6) | 85 | (34.1) |  |  |  |  |  | 29 | (49.2) |  |  |  |  |  |
|  |  |  | 3 points | 0 | (0.0) | 3 | (1.2) |  |  |  |  |  | 1 | (1.7) |  |  |  |  |  |
|  |  | Sleep latency | 0 points | 22 | (48.9) | 76 | (30.5) | 1.82 |  | 1.00 | - | 3.30 | 13 | (22.0) | 3.55 | * | 1.71 | - | 7.35 |
|  |  |  | 1 point | 13 | (28.9) | 107 | (43.0) |  |  |  |  |  | 20 | (33.9) |  |  |  |  |  |
|  |  |  | 2 points | 8 | (17.8) | 50 | (20.1) |  |  |  |  |  | 17 | (28.8) |  |  |  |  |  |
|  |  |  | 3 points | 2 | (4.4) | 16 | (6.4) |  |  |  |  |  | 9 | (15.3) |  |  |  |  |  |
|  |  | Sleep duration | 0 points | 14 | (31.1) | 68 | (27.3) | 0.64 |  | 0.32 | - | 1.31 | 13 | (22.0) | 0.89 |  | 0.38 | - | 2.08 |
|  |  |  | 1 point | 21 | (46.7) | 101 | (40.6) |  |  |  |  |  | 23 | (39.0) |  |  |  |  |  |
|  |  |  | 2 points | 9 | (20.0) | 76 | (30.5) |  |  |  |  |  | 21 | (35.6) |  |  |  |  |  |
|  |  |  | 3 points | 1 | (2.2) | 4 | (1.6) |  |  |  |  |  | 2 | (3.4) |  |  |  |  |  |
|  |  | Sleep efficiency | 0 points | 39 | (86.7) | 191 | (76.7) | 2.06 |  | 0.83 | - | 5.13 | 39 | (66.1) | 3.41 | * | 1.23 | - | 9.41 |
|  |  |  | 1 point | 5 | (11.1) | 43 | (17.3) |  |  |  |  |  | 15 | (25.4) |  |  |  |  |  |
|  |  |  | 2 points | 1 | (2.2) | 13 | (5.2) |  |  |  |  |  | 3 | (5.1) |  |  |  |  |  |
|  |  |  | 3 points | 0 | (0.0) | 2 | (0.8) |  |  |  |  |  | 2 | (3.4) |  |  |  |  |  |
|  |  | Sleep disturbance | 0 points | 16 | (35.6) | 84 | (33.7) | 1.09 |  | 0.56 | - | 2.12 | 20 | (33.9) | 1.14 |  | 0.50 | - | 2.57 |
|  |  |  | 1 point | 28 | (62.2) | 162 | (65.1) |  |  |  |  |  | 38 | (64.4) |  |  |  |  |  |
|  |  |  | 2 points | 1 | (2.2) | 3 | (1.2) |  |  |  |  |  | 1 | (1.7) |  |  |  |  |  |
|  |  | Use of sleep medication | 0 points | 44 | (97.8) | 246 | (98.8) | 0.48 |  | 0.05 | - | 4.76 | 59 | (100.0) | 0.00 |  | 0.00 |  |  |
|  |  |  | 1 point | 0 | (0.0) | 1 | (0.4) |  |  |  |  |  | 0 | (0.0) |  |  |  |  |  |
|  |  |  | 2 points | 1 | (2.2) | 2 | (0.8) |  |  |  |  |  | 0 | (0.0) |  |  |  |  |  |
|  |  | Daytime dysfunction | 0 points | 25 | (55.6) | 118 | (47.4) | 1.41 |  | 0.76 | - | 2.63 | 21 | (35.6) | 2.08 |  | 0.98 | - | 4.42 |
|  |  |  | 1 point | 16 | (35.6) | 101 | (40.6) |  |  |  |  |  | 31 | (52.5) |  |  |  |  |  |
|  |  |  | 2 points | 4 | (8.9) | 26 | (10.4) |  |  |  |  |  | 4 | (6.8) |  |  |  |  |  |
|  |  |  | 3 points | 0 | (0.0) | 4 | (1.6) |  |  |  |  |  | 3 | (5.1) |  |  |  |  |  |
| Team sports | | |  |  |  |  |  |  |  |  |  |  |  |  |  |  |  |  |  |
|  | Subjective sleep quality | | Absence | 23 | (79.3) | 156 | (66.1) | 1.98 |  | 0.77 | - | 5.07 | 24 | (51.1) | 3.73 | * | 1.28 | - | 10.87 |
|  |  |  | Presence | 6 | (20.7) | 80 | (33.9) |  |  |  |  |  | 23 | (48.9) |  |  |  |  |  |
|  | Component scores^a^ | |  |  |  |  |  |  |  |  |  |  |  |  |  |  |  |  |  |
|  |  | Sleep quality | 0 points | 4 | (13.8) | 30 | (12.7) | 1.12 |  | 0.52 | - | 2.39 | 1 | (2.1) | 1.95 |  | 0.79 | - | 4.83 |
|  |  |  | 1 point | 17 | (58.6) | 133 | (56.4) |  |  |  |  |  | 28 | (59.6) |  |  |  |  |  |
|  |  |  | 2 points | 7 | (24.1) | 66 | (28.0) |  |  |  |  |  | 14 | (29.8) |  |  |  |  |  |
|  |  |  | 3 points | 1 | (3.4) | 7 | (3.0) |  |  |  |  |  | 4 | (8.5) |  |  |  |  |  |
|  |  | Sleep latency | 0 points | 15 | (51.7) | 87 | (36.9) | 2.01 |  | 0.95 | - | 4.21 | 12 | (25.5) | 4.24 | * | 1.75 | - | 10.28 |
|  |  |  | 1 point | 11 | (37.9) | 90 | (38.1) |  |  |  |  |  | 15 | (31.9) |  |  |  |  |  |
|  |  |  | 2 points | 3 | (10.3) | 47 | (19.9) |  |  |  |  |  | 14 | (29.8) |  |  |  |  |  |
|  |  |  | 3 points | 0 | (0.0) | 12 | (5.1) |  |  |  |  |  | 6 | (12.8) |  |  |  |  |  |
|  |  | Sleep duration | 0 points | 7 | (24.1) | 75 | (31.8) | 0.64 |  | 0.32 | - | 1.31 | 13 | (27.7) | 0.89 |  | 0.38 | - | 2.08 |
|  |  |  | 1 point | 9 | (31.0) | 87 | (36.9) |  |  |  |  |  | 15 | (31.9) |  |  |  |  |  |
|  |  |  | 2 points | 12 | (41.4) | 67 | (28.4) |  |  |  |  |  | 17 | (36.2) |  |  |  |  |  |
|  |  |  | 3 points | 1 | (3.4) | 7 | (3.0) |  |  |  |  |  | 2 | (4.3) |  |  |  |  |  |
|  |  | Sleep efficiency | 0 points | 23 | (79.3) | 179 | (75.8) | 1.23 |  | 0.48 | - | 3.14 | 32 | (68.1) | 1.94 |  | 0.66 | - | 5.69 |
|  |  |  | 1 point | 4 | (13.8) | 45 | (19.1) |  |  |  |  |  | 10 | (21.3) |  |  |  |  |  |
|  |  |  | 2 points | 2 | (6.9) | 8 | (3.4) |  |  |  |  |  | 3 | (6.4) |  |  |  |  |  |
|  |  |  | 3 points | 0 | (0.0) | 4 | (1.7) |  |  |  |  |  | 2 | (4.3) |  |  |  |  |  |
|  |  | Sleep disturbance | 0 points | 18 | (62.1) | 88 | (37.3) | 2.74 |  | 1.24 | - | 6.08 | 19 | (40.4) | 2.42 |  | 0.94 | - | 6.25 |
|  |  |  | 1 point | 11 | (37.9) | 143 | (60.6) |  |  |  |  |  | 27 | (57.4) |  |  |  |  |  |
|  |  |  | 2 points | 0 | (0.0) | 5 | (2.1) |  |  |  |  |  | 1 | (2.1) |  |  |  |  |  |
|  |  | Use of sleep medication | 0 points | 28 | (96.6) | 230 | (97.5) | 0.83 |  | 0.09 | - | 7.52 | 47 | (100.0) | 0.00 |  | 0.00 |  |  |
|  |  |  | 1 point | 1 | (3.4) | 4 | (1.7) |  |  |  |  |  | 0 | (0.0) |  |  |  |  |  |
|  |  |  | 2 points | 0 | (0.0) | 2 | (0.8) |  |  |  |  |  | 0 | (0.0) |  |  |  |  |  |
|  |  | Daytime dysfunction | 0 points | 18 | (62.1) | 121 | (51.3) | 1.46 |  | 0.67 | - | 3.18 | 19 | (40.4) | 2.47 |  | 0.98 | - | 6.24 |
|  |  |  | 1 point | 9 | (31.0) | 101 | (42.8) |  |  |  |  |  | 22 | (46.8) |  |  |  |  |  |
|  |  |  | 2 points | 1 | (3.4) | 13 | (5.5) |  |  |  |  |  | 6 | (12.8) |  |  |  |  |  |
|  |  |  | 3 points | 1 | (3.4) | 1 | (0.4) |  |  |  |  |  | 0 | (0.0) |  |  |  |  |  |

AOR: adjusted odds ratio, CI: confidence interval

Analysis adjusted for age and sex.

*: P-value was statistically significant.

^a^: P-value was adjusted by Benjamini-Hochberg false discovery rate (FDR) correction

**Supplementary Table 4** Comparison of lifestyle habits between the chronotypes by sports disciplines

|  |  |  | Morningness | | Intermediate | | | | | | | Eveningness | | | | | | |
| --- | --- | --- | --- | --- | --- | --- | --- | --- | --- | --- | --- | --- | --- | --- | --- | --- | --- | --- |
|  |  |  | N | (%) | N | (%) | AOR |  | 95%CI | | | N | (%) | AOR |  | 95%CI | | |
| Individual sports | |  |  |  |  |  |  |  |  |  |  |  |  |  |  |  |  |  |
|  | Skipping breakfast | Yes | 6 | (13.3) | 143 | (57.4) | 9.48 | * | 3.83 | - | 23.46 | 54 | (91.5) | 78.66 | * | 22.11 | - | 279.85 |
|  |  | No | 39 | (86.7) | 106 | (42.6) |  |  |  |  |  | 5 | (8.5) |  |  |  |  |  |
|  | Taking caffeinated drinks | Yes | 16 | (35.6) | 124 | (49.8) | 1.81 |  | 0.93 | - | 3.49 | 25 | (42.4) | 1.34 |  | 0.60 | - | 2.98 |
|  |  | No | 29 | (64.4) | 125 | (50.2) |  |  |  |  |  | 34 | (57.6) |  |  |  |  |  |
|  | Use of smartphone/cellphone after lights out | Yes | 23 | (51.1) | 167 | (67.1) | 1.94 |  | 1.02 | - | 3.70 | 48 | (81.4) | 4.25 | * | 1.76 | - | 10.25 |
|  |  | No | 22 | (48.9) | 82 | (32.9) |  |  |  |  |  | 11 | (18.6) |  |  |  |  |  |
|  | Morning practice | 4–7 days/week | 17 | (37.8) | 48 | (19.3) | 0.38 | * | 0.19 | - | 0.76 | 3 | (5.1) | 0.09 | * | 0.02 | - | 0.32 |
|  |  | 0–3 days/week | 28 | (62.2) | 201 | (80.7) |  |  |  |  |  | 56 | (94.9) |  |  |  |  |  |
| Team sports | |  |  |  |  |  |  |  |  |  |  |  |  |  |  |  |  |  |
|  | Skipping breakfast | Yes | 7 | (24.1) | 118 | (50.0) | 3.18 | * | 1.30 | - | 7.77 | 33 | (70.2) | 7.71 | * | 2.66 | - | 22.35 |
|  |  | No | 22 | (75.9) | 118 | (50.0) |  |  |  |  |  | 14 | (29.8) |  |  |  |  |  |
|  | Taking caffeinated drinks | Yes | 11 | (37.9) | 111 | (47.0) | 1.52 |  | 0.68 | - | 3.37 | 25 | (53.2) | 1.96 |  | 0.75 | - | 5.08 |
|  |  | No | 18 | (62.1) | 125 | (53.0) |  |  |  |  |  | 22 | (46.8) |  |  |  |  |  |
|  | Use of smartphone/cellphone after lights out | Yes | 14 | (48.3) | 166 | (70.3) | 2.71 | * | 1.23 | - | 5.96 | 36 | (76.6) | 3.81 | * | 1.40 | - | 10.40 |
|  |  | No | 15 | (51.7) | 70 | (29.7) |  |  |  |  |  | 11 | (23.4) |  |  |  |  |  |
|  | Morning practice | 4–7 days/week | 9 | (31.0) | 55 | (23.3) | 0.69 |  | 0.30 | - | 1.61 | 3 | (6.4) | 0.16 | * | 0.04 | - | 0.64 |
|  |  | 0–3 days/week | 20 | (69.0) | 181 | (76.7) |  |  |  |  |  | 44 | (93.6) |  |  |  |  |  |

AOR: adjusted odds ratio, CI: confidence interval

Analysis adjusted for age and sex.

* P value was statistically significant after applying the Benjamini–Hochberg false discovery rate (FDR) correction.

**Supplementary Table 5** Relationships between morning practice and subjective sleep quality across chronotypes and sports disciplines

|  |  |  | Morningness | | | |  |  | Intermediate | | | |  |  | Eveningness | | | |  |  |
| --- | --- | --- | --- | --- | --- | --- | --- | --- | --- | --- | --- | --- | --- | --- | --- | --- | --- | --- | --- | --- |
|  |  |  | Subjective sleep quality | | | | | | | | | | | | | | | | | |
|  |  |  | Poor | | Good | |  |  | Poor | | Good | |  |  | Poor | | Good | |  |  |
|  |  |  | N | (%) | N | (%) | *P* |  | N | (%) | N | (%) | *P* |  | N | (%) | N | (%) | *P* |  |
| Individual sports | |  |  |  |  |  |  |  |  |  |  |  |  |  |  |  |  |  |  |  |
|  | Morning practice | 0–3 days/week | 6 | (54.5) | 22 | (64.7) | 0.722 | ^a^ | 76 | (79.2) | 125 | (81.7) | 0.622 | ^b^ | 33 | (91.7) | 23 | (100.0) | 0.274 | ^a^ |
|  |  | 4–7 days/week | 5 | (45.5) | 12 | (35.3) |  |  | 20 | (20.8) | 28 | (18.3) |  |  | 3 | (8.3) | 0 | (0.0) |  |  |
| Team sports | |  |  |  |  |  |  |  |  |  |  |  |  |  |  |  |  |  |  |  |
|  | Morning practice | 0–3 days/week | 5 | (83.3) | 15 | (65.2) | 0.633 | ^a^ | 63 | (78.8) | 118 | (75.6) | 0.593 | ^b^ | 20 | (87.0) | 24 | (100.0) | 0.109 | ^a^ |
|  |  | 4–7 days/week | 1 | (16.7) | 8 | (34.8) |  |  | 17 | (21.3) | 38 | (24.4) |  |  | 3 | (13.0) | 0 | (0.0) |  |  |

^a^ Fisher’s exact test

^b^ Chi-square test
